# Factors associated with whole carcass condemnation rates in provincially-inspected abattoirs in Ontario 2001-2007: implications for food animal syndromic surveillance

**DOI:** 10.1186/1746-6148-6-42

**Published:** 2010-08-12

**Authors:** Gillian D Alton, David L Pearl, Ken G Bateman, W Bruce McNab, Olaf Berke

**Affiliations:** 1 Department of Population Medicine, Ontario Veterinary College, University of Guelph, Guelph, Ontario, N1G 2W1, Canada; 2 Ontario Ministry of Agriculture, Food, and Rural Affairs, Guelph, Ontario, N1G 4Y2, Canada

## Abstract

**Background:**

Ontario provincial abattoirs have the potential to be important sources of syndromic surveillance data for emerging diseases of concern to animal health, public health and food safety. The objectives of this study were to: (1) describe provincially inspected abattoirs processing cattle in Ontario in terms of the number of abattoirs, the number of weeks abattoirs process cattle, geographical distribution, types of whole carcass condemnations reported, and the distance animals are shipped for slaughter; and (2) identify various seasonal, secular, disease and non-disease factors that might bias the results of quantitative methods, such as cluster detection methods, used for food animal syndromic surveillance.

**Results:**

Data were collected from the Ontario Ministry of Agriculture, Food and Rural Affairs and the Ontario Cattlemen's Association regarding whole carcass condemnation rates for cattle animal classes, abattoir compliance ratings, and the monthly sales-yard price for various cattle classes from 2001-2007. To analyze the association between condemnation rates and potential explanatory variables including abattoir characteristics, season, year and commodity price, as well as animal class, negative binomial regression models were fit using generalized estimating equations (GEE) to account for autocorrelation among observations from the same abattoir. Results of the fitted model found animal class, year, season, price, and audit rating are associated with condemnation rates in Ontario abattoirs. In addition, a subset of data was used to estimate the average distance cattle are shipped to Ontario provincial abattoirs. The median distance from the farm to the abattoir was approximately 82 km, and 75% of cattle were shipped less than 100 km.

**Conclusions:**

The results suggest that secular and seasonal trends, as well as some non-disease factors will need to be corrected for when applying quantitative methods for syndromic surveillance involving these data. This study also demonstrated that animals shipped to Ontario provincial abattoirs come from relatively local farms, which is important when considering the use of spatial surveillance methods for these data.

## Background

The monitoring and surveillance of emerging infectious and zoonotic diseases in food animals are important components of our food safety system. In recent years, emerging zoonotic diseases have been of increased concern to both public and animal health, following the emergence of H5N1 influenza and bovine spongiform encephalopathy (BSE) [1]. Consequently, researchers are turning their attention to novel approaches, such as syndromic surveillance, for detecting emerging diseases in food animals at various points along the farm-to-fork continuum [2,3].

Abattoirs have played an important role in the surveillance of various diseases of human and animal health importance [4-6]. Surveillance at the abattoir allows for all animals passing into the human food chain to be examined for unusual signs, lesions or specific diseases. For instance, a study evaluating surveillance systems for bovine tuberculosis in Switzerland found that surveillance during meat inspection at the slaughterhouse had the highest sensitivity for identifying the disease compared to passive clinical surveillance of humans or cattle on farms [5]. A relatively new application of surveillance methods for animal health, food safety and public health is syndromic surveillance of food animals.

Syndromic surveillance is the grouping of large numbers of signs/symptoms and data regarding non-traditional sources of information. These groups of signs/symptoms are loosely designated as 'syndromes'. This information is then used to track disease trends in a population and signal putative outbreaks that warrant further investigation [7]. Syndromic surveillance has been primarily used in public health practice [8-10] and has had some success at the early detection of disease outbreaks in humans [11]. Recently, syndromic surveillance has been applied to animal health using data from farms, sales-yards, veterinary practitioners, and abattoir condemnation data [2,3,12]. Changes in the incidence of lesions at slaughter may provide important information for syndromic surveillance of diseases of animal, public health, and food safety significance.

The application of appropriate quantitative methods is important for any surveillance system. A variety of statistical methods has been developed and is frequently used for disease surveillance, including spatial, temporal and spatio-temporal methods [7]. In general, these methods use various statistical detection algorithms to analyze a continuous stream of data and raise an alarm when the count is significantly greater than expected, suggesting a possible disease outbreak [13]. Depending on the method used and the data collected, one might be able to identify the area and/or time of the disease outbreak. Although, these methods have been shown to be useful, surveillance systems are only as good as the data provided to the system. Consideration of the quality of the data and naturally occurring covariates need to be taken into account in the selection, application and interpretation of quantitative methods for disease surveillance.

Recent literature has suggested the need for model-based approaches for surveillance in order to include other variables into the specification of expected disease incidence [14]. For many diseases, the incidence may vary with biological factors such as sex and season. In addition, factors associated with the reporting of disease may also impact the apparent incidence of disease. For example, price of the commodity may be associated with the quality of animals being shipped to slaughter, which then in turn will affect the condemnation rate in abattoirs. Being able to account for these known factors prior to the application of cluster detection methods, may improve the sensitivity and specificity of a quantitative syndromic surveillance system [15,16]. The quality of spatial data is also important to consider prior to the application of space or space-time cluster detection methods [17]. These issues may be particularly true for Ontario provincial abattoir data where factors, such as the capacity of an abattoir may be correlated with the quality of animals they receive, and the location of the abattoir can only approximately reflect the spatial location of an animal's farm of origin.

It was hypothesized that a variety of factors may be associated with the condemnation rates seen in abattoirs, and that these effects may vary in space and/or time. Abattoir characteristics, such as the number of weeks abattoirs processed animals and the number of animals processed, may be associated with condemnation rates, as the speed of processing may impact inspection. An abattoir's audit rating may reflect a plant's compliance to regulations and/or willingness to accept animals of poorer quality. Region of the abattoir may be associated with condemnation rates in provincial abattoirs due to regional differences in animal density and disease prevalence. Season and year may also be associated with condemnation rates as many diseases in animals are thought to have seasonal and secular variability. Animal class may be associated with condemnation rates, as older animals are generally at higher risk of disease. Economic factors, such as commodity price fluctuate greatly and may be associated with the quality of animals being sent to slaughter, as producers consider shipping costs against the possible return of shipping an animal of suspect health.

Consequently, the objectives of this study were to identify biological and non-biological factors that may be associated with abattoir condemnations and possibly influence cluster detection methods for quantitative syndromic surveillance systems. Specifically, provincially inspected abattoirs which slaughter cattle in Ontario were characterized in terms of number of abattoirs, the number of weeks abattoirs processed cattle, geographical distribution, types of condemnations reported, and distance animals are shipped to provincial abattoirs. Secondly, this study will determine how abattoir characteristics, season, year and commodity price, and animal class may be associated with whole carcass condemnation rates in provincial abattoirs. In addition, the suitability of Ontario provincial abattoir data for spatial and spatio-temporal analyses will be considered based on the results.

## Methods

### Data source and variables

Whole carcass condemnation data were obtained from the Food Safety Decision Support System (FSDSS) database maintained by the Ontario Ministry of Agriculture, Food and Rural Affairs (OMAFRA). Data were extracted from the database for cattle animal classes: bulls, calves, cows, heifers and steers from January 1, 2001 to December 31, 2007. Missing geographical coordinates for abattoirs were approximated using postal codes and/or addresses with the address geocoding software GeoPinpoint Suite 6.4 (DMTI Spatial Inc., Markham, Ontario, Canada). Using the FSDSS database, the following information was extracted for each month: abattoir identification number, geographical coordinates of abattoir, year, season, number of weeks an abattoir was open each year, total number of whole carcasses condemned, total number of cattle processed each year, and animal class. Season was categorized by 3 month groupings as follows: winter (December - February), spring (March - May), summer (June - August) and fall (September - November). Animal class included five categories: bulls, cows, calves, heifers, and steers. Bulls were excluded from subsequent statistical analyses due to missing data and inconsistencies in the use of this classification. The number of weeks an abattoir was open each year was determined by the total number of weeks in which at least one bovine animal was processed. The total number of animals processed each year was calculated by adding the total number of condemned cattle and the total number of cattle fit for consumption each year. Linearity of continuous variables was assessed by plotting the log of the condemnation rate against the covariate using a lowess smoother. If there was no visible linear relationship between the outcome and the covariate, and the association could not be adequately modeled with a quadratic term, or transformation, then the variable was categorized.

Abattoir audit ratings were obtained for all abattoirs from the abattoir audit program administered through OMAFRA. The audit program assesses each facility's food safety performance and compliance with the Ontario Meat Inspection Act. Audits are conducted once a year and evaluate each premise on 14 food safety areas based on the Standards of Compliance relating to food safety, animal welfare and occupational health and safety with a letter grade given for each abattoir [18]. Annual OMAFRA audit ratings were obtained for all abattoirs in the audit program from 2001-2007. Abattoir audit ratings were classified according to the letter grade received from best to poorest as follows: AAA, AA, A, B or C and unrated for abattoirs that had missing data.

The price of cattle was obtained from the Ontario Cattlemen's Association market reports for 2001-2007. Prices were calculated to be the average price (in Canadian dollars) per lbs based on sales records from Ontario sales-yards. A price was assigned for each month and year by animal class. The most appropriate weight category was selected to represent each animal class based on an average animal at the time of slaughter.

The agricultural region where the abattoir was located was classified as: central, eastern, northern, southern or western Ontario using the Census Agricultural Region boundaries (Statistics Canada, Census Agricultural Regions, Census year 2001). The regional location of each abattoir was determined using the point-in-polygon technique with geographic information system software ArcGIS 9.2 (ESRI, Redlands, California, USA).

Travel distance between the animals' farm and the abattoir was estimated using data obtained from OMAFRA. Because farm location is not routinely recorded with condemnation data, a subset of cattle, in which a sample was sent for laboratory testing, were used to obtain geo-location information for the abattoir and farm. Like abattoir location, owner address information was geo-coded according to the owner postal code using geocoding software GeoPinpoint Canada. Distance from the farm to abattoir was calculated using the Haversine distance formula, which calculates the great-circle distances between two points on a sphere using their longitudes and latitudes [19].

Data from all sources were merged into one master dataset using Stata 10.1 (Stata Corp., College Station, Texas, USA).

### Statistical analysis

To model and evaluate their association with monthly whole carcass cattle condemnation rates, the effect of year, season, annual audit rating, number of weeks open, number of cattle processed, census agricultural region, animal class and sales price of animal class were included in the model. All covariates were evaluated for statistical significance individually and then in a multivariable model using generalized estimating equations (GEE) to fit a negative binomial regression model with an exchangeable correlation structure to account for repeated measurements from each abattoir. Wald tests were performed on each covariate in the model to estimate the significance of each categorical variable as a group. Non-significant covariates (p ≥ 0.05) based on the Wald test were removed from the model. All excluded covariates were evaluated for their potential confounding effect by evaluating if their removal produced a 20% or greater change in the coefficient of significant variables in the model. Interactions between price and year, year and number of animals processed, year and animal class, as well as season and animal class were investigated. In addition, the covariates included in the model were then fitted using GEE with both Poisson and negative binomial distributions and the following correlation structures: exchangeable, first order autoregressive structure, second order autoregressive structure, non-stationary, and stationary. All resulting models were evaluated for how well the model fit the data using a quasi-log-likelihood under the independence model information criterion (QIC) statistic for model selection [20]. The model with the lowest QIC was selected as the final model. Robust standard errors were used for all GEE fitted models. All statistical analyses were performed using Stata 10.1.

## Results

### Descriptive statistics

**Figure 1:**
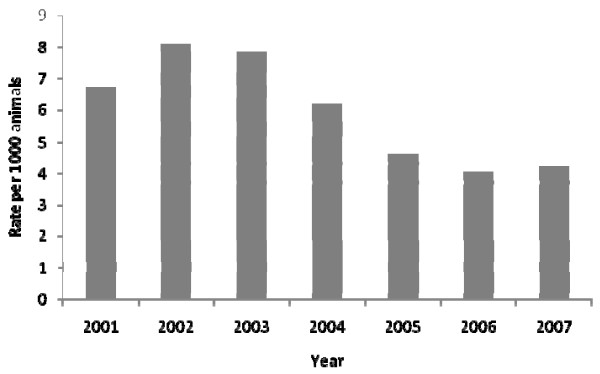
**Condemnation rates per 1000 cattle from Ontario provincial abattoirs 2001-2007**. Whole carcass condemnation rates per 1000 cattle from Ontario provincial abattoirs 2001-2007.

**Figure 2:**
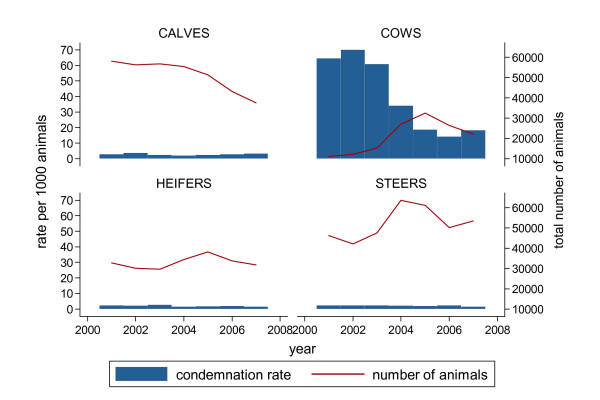
**Animal class condemnation rates per 1000 cattle from Ontario provincial abattoirs 2001-2007**. Condemnation rates per 1000 cattle and total number of cattle in each animal class from Ontario provincial abattoirs 2001-2007.

There were 207 provincially-inspected abattoirs processing a total of 1,162,410 cattle from 2001 - 2007 with the following animal class distribution: 5.4% bulls, 12.6% cows, 19.8% heifers, 30.8% calves and 31.3% steers. The number of abattoirs processing at least one bovine animal each year varied over the study period (Table 1). Of the total number of processed cattle, 6875 carcasses were condemned for various reasons with septicaemia and/or toxaemia being typically the most common reason for condemnation (Table 2). The condemnation rate per 1000 animals fluctuated over the study period with the most prominent decrease occurring during 2004-2005 (Figure 1). Average overall condemnation rates were much greater in cows compared to other cattle classes (Figure 2). However, the overall decreasing trend in condemnation rates was most marked in cows in 2006 (Figure 2). The total number of animals being processed peaked in cows, heifer and steers at some point during the 2004-2006 period (Figure 2).

**Table 1:** Number of provincially inspected abattoirs in Ontario 2001-2007

Year	Number of abattoirs reporting bovine carcass counts
2001	163
2002	158
2003	147
2004	143
2005	148
2006	139
2007	129

Total	207

Number of provincially inspected abattoirs in Ontario processing at least one bovine animal per year and the total number of provincially inspected abattoirs processing cattle in Ontario from 2001-2007.

**Table 2:** Reason for whole carcass condemnation in provincially inspected abattoirs in Ontario 2001-2007

Condemnation reason	Percentage of total yearly condemnations						
	**Year**						
	**2001**	**2002**	**2003**	**2004**	**2005**	**2006**	**2007**
	
Abscess	7.4	9.4	7.3	8.8	9.8	10.5	9.8
Animal arrived dead^1^	0	0	2.7	0.4	1.1	0.8	0.6
Animal found dead^2^	4.7	5.8	4.9	4.4	7.5	7.2	6.8
Arthritis	3.5	2.4	2.9	2.9	1.9	4.0	3.2
Bruising	9.1	9.8	7.8	3.0	2.0	1.4	1.1
Chemical Residue	0	0.1	0.0	0.0	0.0	0.0	0.0
Contamination	0.1	0.1	0.2	0.1	0.2	0.2	0.3
Cystericercus bovis	0.0	0	0.0	0.2	0.0	0.0	0.0
Drug residue	3.0	2.0	1.5	0.0	0.1	0.3	3.1
Edema	2.3	3.7	3.4	3.4	1.3	1.2	0.8
Emaciation	6.6	6.0	7.7	6.6	10.7	8.4	9.7
Icterus	0.6	1.2	1.5	1.6	1.7	1.2	1.5
Inadequate bleeding	0.4	0.1	0.9	3.0	1.5	0.5	0.8
Lymphadenitis	1.5	2.2	5.3	6.7	4.5	4.6	3.4
Lymphosarcoma neoplasm	12.6	13.1	12.4	6.6	4.4	2.7	4.3
Mastitis	0.0	0.1	0.2	0.2	0.6	0.2	0.3
Metritis	0.4	0.1	0.1	0.4	0.7	0.2	0.3
Moribund	1.4	1.3	0.7	1.0	1.1	0.9	1.8
Myositis	0.7	0.2	0.2	0.2	0.6	1.1	0.8
Neoplasm other	3.0	1.9	2.7	1.8	2.1	3.0	2.5
Nephritis	0.8	0.7	0.5	0.3	1.2	0.9	0.5
Odour	2.2	1.2	1.5	0.8	0.3	0.2	0.2
Operator requested condemnation	0.0	0.0	0.0	4.5	10.6	7.9	8.9
Other disease	6.9	6.2	5.3	8.7	5.1	4.9	3.4
Pericarditis	2.0	1.7	1.1	1.3	1.9	2.1	1.7
Peritonitis	6.5	6.2	6.0	6.1	6.1	7.6	6.9
Pleuritis	0.4	0.8	0.6	0.5	1.3	1.4	0.6
Pneumonia	2.7	3.1	2.2	4.1	5.5	4.6	4.8
Pyelonephritis	0.3	0.2	0.9	0.0	1.2	1.4	2.5
Rabies	0.0	0.1	0.0	0.0	0.0	0.0	0.0
Septicaemia and/or toxemia	11.7	15.6	15.0	17.7	11.6	14.0	15.8
Squamous cell carcinoma neoplasm	1.2	0.4	0.9	1.3	0.8	1.1	1.1
Toxaemia	6.0	3.2	2.5	2.3	0.7	4.4	0.5
Uremia	2.3	1.2	1.5	1.3	1.8	1.4	2.2

Total number of condemnations per year	1047	1203	1236	1188	895	656	650

^1 ^Refers to animals which are dead when the truck pulls into the plant premises

^2 ^Inspector finds a dead animal in the pen that had arrived alive during ante-mortem inspection in the barn

Reason and percentage of whole cattle carcasses condemned in provincially inspected abattoirs in Ontario from 2001-2007.

The quartiles of total number of animals and the corresponding number of processing abattoirs were tabulated for each year of the study period (Table 3a). With the exception of 2004, 2005 and 2007, most of the abattoirs processed fewer than 500 cattle per year. The quartiles of the total number of weeks each year an abattoir processed at least one animal and the corresponding number of abattoirs was tabulated for each year of the study period (Table 3b). Over the study period, there was an increasing trend in the number of abattoirs processing cattle more than 49 weeks per year. On average, approximately 1.5% of abattoirs processed cattle only 1 week per year, 7% processed cattle a quarter of the year or less, and 20% processed cattle up to half of the year. During the study period only 19% of abattoirs processed cattle 52 weeks per year. The annual OMAFRA audit rating scores and the corresponding number of abattoirs receiving those scores are shown in Table 3c for each year during the study period. Throughout this period, the majority of rated abattoirs were given an "A" rating. The median sales price of each animal class was calculated for each year during the study period (Table 3d). The median sales-price in all cattle classes was lowest in 2004. No continuous variables were found to have a linear relationship with cattle carcass condemnation rates, therefore, quartiles of the empirical distribution were used to categorize the total number of animals processed and the number of weeks an abattoir was open (Table 3a and Table 3b, respectively). Price was categorized into a dichotomous variable according to the yearly median sales price for each animal class (Table 3d).

**Table 3:** Summary of number of cattle processed, number weeks open, audit rating and animal class

a) Number of animals processed	Number of Abattoirs						
	2001	2002	2003	2004	2005	2006	2007
1 - 286	73	72	36	24	29	40	38
287 - 498	42	35	40	28	34	34	25
499 - 841	25	24	36	40	43	32	36
842 - 27 286	22	26	33	49	41	32	30

**b) Number of weeks open**	**Number of Abattoirs**						

1 - 43	66	67	44	32	43	38	38
44 - 49	58	46	48	45	30	39	29
50 - 51	17	27	25	31	41	31	32
52	21	17	28	33	33	30	30

**c) Audit Rating**	**Number of Abattoirs**						

AAA	1	0	0	0	0	1	1
AA	6	6	7	13	16	23	28
A	61	72	79	78	78	78	72
B	15	8	8	5	8	6	9
C	2	1	0	0	1	0	0
Unrated	78	71	53	47	45	31	19

**d) Animal Class**	**Median sales-price of animal class per year 2001-2007**						

Calves	136.53	117.95	112.19	83.61	107.14	119.69	108.49
Cows	64.28	58.64	27.11	19.70	25.38	31.84	34.77
Heifers	112.01	101.22	82.16	73.61	87.74	90.87	91.09
Steers	113.24	102.57	83.15	75.60	91.07	93.55	92.56

Summary of the a) quartiles of the total number of cattle processed, b) quartiles of the number of weeks at least one bovine animal was processed, c) annual OMAFRA audit rating, and d) median sales price of calves, cows, heifers and steers 2001-2007.

**Figure 3:**
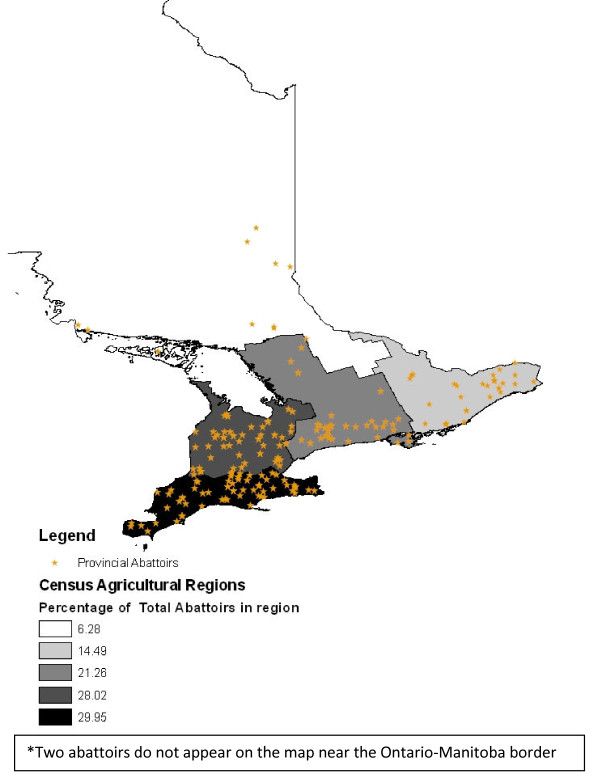
**Choropleth map of percentage of abattoirs processing cattle in Ontario per census agricultural region**. Choropleth map of the percentage of provincially inspected abattoirs processing cattle in Ontario in each census agricultural region and the location of each abattoir 2001-2007.

Provincial abattoirs are located throughout Ontario with the majority situated in southern and western Ontario and fewest located in northern Ontario (Figure 3). The distance between abattoir and farm was calculated for 2456 samples sent for laboratory testing from 107 of the 207 abattoirs processing cattle from 2001-2007. Results indicated that the median distance between the farm and abattoir was 82 km, with 25% of all farms located within 34 km of the abattoir, and 75% within 94 km of the abattoir.

### Statistical models

Results of the univariable GEE modeling approach indicated that animal class (χ^2 ^= 147.39, p < 0.001), region (χ^2 ^= 12.83, p = 0.012), audit rating (χ^2 = ^351.23, p < 0.001), season (χ^2 ^= 10.41, p = 0.015), and price (χ^2 ^= 4.05, p = 0.044) all had statistically significant associations with the outcome according to the Wald test performed to determine the significance of the entire variable in the model (Table 4). Year, the number of weeks an abattoir was open, and the total number of animals processed were not significant based on Wald tests (χ^2 ^= 11.82, p = 0.066; χ^2 ^= 3.95, p = 0.267; and χ^2 ^= 1.21, p = 0.751, respectively).

**Table 4:** Univariable negative binomial models using GEE^1 ^approach

Covariate	**IRR**^**2**^	Robust Standard Error	P-value	95% CI
Animal class				

Calves	---	---	---	---
Cows	4.83	1.35	< 0.001	2.80 - 8.34
Heifers	0.43	0.09	< 0.001	0.28 - 0.64
Steers	0.51	0.11	0.01	0.34 - 0.77

Year				

2001	---	---	---	---
2002	1.02	0.14	0.90	0.77 - 1.34
2003	0.90	0.14	0.49	0.67 - 1.22
2004	0.70	0.15	0.10	0.46 - 1.06
2005	0.69	0.14	0.07	0.47 - 1.03
2006	0.75	0.16	0.17	0.50 - 1.13
2007	0.73	0.17	0.18	0.46 - 1.16

Season				

Winter	---	---	---	---
Spring	0.94	0.05	0.20	0.85 - 1.03
Summer	0.86	0.06	0.02	0.76 - 0.98
Fall	0.76	0.07	0.01	0.64 - 0.91

^3^Audit rating				

C	---	---	---	---
AAA	0.71	0.53	0.64	0.16 - 3.07
AA	0.90	0.37	0.80	0.40 - 2.01
A	1.19	0.48	0.67	0.54 - 2.61
B	1.01	0.42	0.98	0.45 - 2.28
unrated	2.69	1.09	0.01	1.22 - 5.94

# weeks open				

1 - 43	---	---	---	---
44 - 49	0.72	0.15	0.10	0.48 - 1.08
50 - 51	0.71	0.16	0.13	0.46 - 1.11
52	0.92	0.29	0.78	0.49 - 1.70

# animal processed				

1 - 286	---	---	---	---
287 - 498	1.01	0.15	0.95	0.75 - 1.35
499 - 841	1.14	0.19	0.44	0.82 - 1.58
842 - 27286	1.29	0.42	0.43	0.68 - 2.46

Region				

Central	---	---	---	---
Eastern	9.88	6.83	0.01	2.55 - 38.26
Northern	3.78	2.49	0.04	1.04 - 13.74
Southern	7.91	6.56	0.01	1.56 - 40.21
Western	1.60	2.31	0.75	0.09 - 27.23

Price				

Below median	---	---	---	---
Above median	1.11	0.06	0.04	1.00 - 1.23

^1 ^Generalized estimating equation using an exchangeable correlation structure to accommodate repeated measurements among provincial abattoirs

^2 ^Incidence rate ratio

^3 ^Model would not converge therefore results represent univariable negative binomial model

Univariable negative binomial models using a GEE^1 ^approach modeling the association between condemnation rates in Ontario provincial abattoirs and animal class, year, season, audit rating, the number of weeks an abattoir was open each year, the number of cattle an abattoir processed each year, census agricultural region and median yearly sales-price for animal class.

Multivariable Poisson and negative binomial models fit by GEE were investigated using a variety of correlation structures (Table 5). Based on the QIC statistic, the best fitting model was a multivariable negative binomial regression model using an exchangeable correlation structure. Animal class, year, season, price and audit rating of abattoirs were the only statistically significant variables in the model. The interaction between animal class and year was the only statistically significant interaction term. There was no evidence that excluded variables confounded the remaining variables. The fitted model indicated that during 2005 - 2006 cows had the most prominent decrease in condemnation rates in abattoirs compared to calves in 2001, based on the size of the cows x 2005 and cows x 2006 interaction terms (Table 6). Condemnation rates for cows ranged from approximately 3 to 8 times greater than calves throughout the study period (Table 7). Condemnation rates were significantly lower in heifers during 2002 compared to calves in 2001 (Table 6). In comparison to winter, condemnation rates were significantly lower in the summer and fall (Table 6). Condemnation rates were also significantly higher in C rated abattoirs compared to higher rated abattoirs. Condemnation rates were higher in cattle when the sales price of the animal class was above the yearly median (Table 6).

**Table 5:** Comparison of GEE^1^ fitted models using QIC^2^ statistic

Model	Correlation Structure	QIC
Negative binomial GEE^2^	Exchangeable	11328
	Non-stationary	11626
	2^nd ^order autoregressive	11647
	Stationary	11664
	1^st ^order autoregressive	11668
Poisson GEE^2^	2^nd ^order autoregressive	12007
	Stationary	12056
	1^st ^order autoregressive	12409
	Exchangeable	13605
	Non-stationary	Did not converge

^1^Generalized estimating equation

^2^Quasi-log- likelihood under the independence model information criterion

Comparison of QIC^2 ^statistic values for negative binomial or Poisson models using a GEE^1 ^approach to investigate the association between condemnation rates in Ontario Provincial abattoirs and year, season, animal class, price, year-animal class interaction and audit rating.

**Table 6:** Multivariable negative binomial model using a GEE^1^ approach

Covariate	**IRR**^**2**^	Robust Standard Error	P-value	95% CI
Animal class				

Calves	---	---	---	---
Cows	6.73	2.40	< 0.001	3.35 - 13.52
Heifers	0.47	0.10	< 0.001	0.31 - 0.72
Steers	0.46	0.12	0.01	0.29 - 0.74

Year				

2001	---	---	---	---
2002	1.37	0.21	0.04	1.02 - 1.84
2003	0.70	0.16	0.11	0.45 - 1.09
2004	0.56	0.13	0.02	0.36 - 0.90
2005	0.93	0.20	0.74	0.61 - 1.43
2006	1.05	0.23	0.80	0.69 - 1.63
2007	1.16	0.29	0.56	0.71 - 1.88

Season				

Winter	---	---	---	---
Spring	0.88	0.07	0.07	0.76 - 1.01
Summer	0.82	0.06	0.01	0.71 - 0.95
Fall	0.77	0.05	< 0.001	0.69 - 0.87

Audit rating				

C	---	---	---	---
AAA	0.32	0.30	0.22	0.05 - 2.01
AA	0.43	0.12	0.01	0.25 - 0.74
A	0.64	0.11	0.01	0.46 - 0.89
B	0.56	0.11	0.01	0.39 - 0.82
unrated	1.41	0.35	0.17	0.87 - 2.31

Price				

Below median	---	---	---	---
Above median	1.12	0.05	0.02	1.02 - 1.23

Year x animal class				

Calves and 2001	---	---	---	---
Cows and 2002	0.61	0.12	0.01	0.42 - 0.88
Cows and 2003	1.13	0.34	0.69	0.63 - 2.05
Cows and 2004	0.83	0.30	0.61	0.40 - 1.72
Cows and 2005	0.48	0.14	0.01	0.27 - 0.86
Cows and 2006	0.45	0.12	0.01	0.27 - 0.78
Cows and 2007	0.51	0.19	0.06	0.25 - 1.04
Heifers and 2002	0.61	0.14	0.03	0.39 - 0.95
Heifers and 2003	1.46	0.44	0.21	0.81 - 2.65
Heifers and 2004	1.05	0.30	0.85	0.61 - 1.83
Heifers and 2005	0.88	0.24	0.63	0.51 - 1.50
Heifers and 2006	0.93	0.22	0.77	0.59 - 1.48
Heifers and 2007	0.71	0.23	0.29	0.39 - 1.33
Steers and 2002	0.74	0.14	0.12	0.51 - 1.08
Steers and 2003	1.40	0.43	0.28	0.77 - 2.54
Steers and 2004	1.63	0.50	0.11	0.89 - 2.99
Steers and 2005	1.02	0.28	0.94	0.60 - 1.73
Steers and 2006	1.42	0.44	0.26	0.78 - 2.61
Steers and 2007	0.90	0.32	0.77	0.45 - 1.81

Correlation value = 0.067				

^1 ^Generalized estimating equation using an exchangeable correlation structure to account for repeated measurements among provincial abattoirs

^2 ^Incidence rate ratio

Multivariable negative binomial models using a GEE^1 ^approach investigating the association between condemnation rates in Ontario provincial abattoirs and animal class, year, season, price, year-animal class interaction and audit rating based on the multivariable GEE model.

**Table 7:** Linear combinations of condemnation rates in cows and calves

Contrast	IRR	P-value	95% CI
Cows 2001 vs. Calves 2001	6.73	< 0.001	3.35 - 13.52
Cows 2002 vs. Calves 2002	4.09	< 0.001	2.23 - 7.50
Cows 2003 vs. Calves 2003	7.61	< 0.001	4.00 - 14.46
Cows 2004 vs. Calves 2004	5.56	< 0.001	2.81 - 10.99
Cows 2005 vs. Calves 2005	3.23	< 0.001	1.90 - 5.50
Cows 2006 vs. Calves 2006	3.06	< 0.001	1.86 - 5.01
Cows 2007 vs. Calves 2007	3.41	< 0.001	1.97 - 5.90

Linear combinations of condemnation rates in cows compared to calves in Ontario Provincial abattoirs 2001-2007 based on model in Table 6.

## Discussion

Provincial abattoir data are useful for surveillance because they can provide a more regionally specific picture of emerging diseases in Ontario. However, various biological and non-biological factors were found to have an effect on condemnation rates. Consequently, careful consideration should be given to how these factors may influence quantitative methods designed for outbreak detection. If these variables are ignored, quantitative methods designed to identify trends or disease clusters may not provide valid results.

Results from this study regarding the distance animals are shipped to slaughter indicated that the majority of cattle are shipped less than 100 km. Within the spatial scale of the province of Ontario, which is approximately one million square kilometres [21], we can conclude that cattle sent to provincial abattoirs come from relatively local farms. This result is important for the application of quantitative spatial surveillance methods, as the assumption that abattoir data reflects disease rates among locally slaughtered animals appears to be valid.

Seasonal effects were noted in the results of the multivariable analysis with summer and fall having lower condemnation rates compared to winter. This may be due to a change in the quality of animals being submitted over the year. Animals which are shipped during the winter may reflect those animals which grew slower due to certain health issues causing delayed market readiness, thus, resulting in more condemnations at slaughter during this season. More research on quality of animals and point in production cycle is needed to confirm if this trend reflects "poor-doers". It is important to identify seasonal trends before the application of quantitative surveillance methods, as many diseases have seasonal variability, and without correcting for this trend, any results from temporal or spatial-temporal quantitative methods will be biased.

Commodity class and year were found to be associated with condemnation rates in provincial abattoirs. It is suspected that the discovery of BSE in Alberta, Canada in May 2003 [22] had an impact on the patterns of condemnations in Ontario provincial abattoirs. Within hours of the confirmation of the first Canadian case, the United States (US) government announced an immediate ban of all imports of Canadian beef. On July 24, 2003, new processing regulations were implemented in Canadian abattoirs outlining that specified risk materials (SRMs), such as brain and spinal cord, must be removed from cattle older than 30 months. After 26 months, the US ban on Canadian cattle imports was lifted [23]. The effects of BSE in Canada, and subsequent changes to trade and processing regulations are mirrored in the descriptive data, which showed a decreasing trend over the study period in the number of abattoirs processing cattle, as well as overall condemnation rates and condemnation rates by animal class. The decreasing trend levels off in 2004 - 2006 where rates begin to increase again. Condemnation rates in cows showed the largest drop, coinciding with regulations implemented for cattle over 30 months [22]. These trends are also mirrored in the interaction effect between year and animal class seen in the multivariable model. Condemnation rates for cows ranged from approximately 3 to 8 times greater than calves throughout the study period, with cows during 2005 - 2006 having the most prominent decrease in condemnation rates in abattoirs compared to calves in 2001. Being older, cows are at higher risk for disease and thus are condemned more frequently. The decline in cow condemnation rates during this period occurred while the total number of cows being processed by provincial abattoirs increased. In the case of cows, the decline in the rate of condemnations most likely resulted from younger and healthier cows being shipped for slaughter to provincial abattoirs, which ship their products intra-provincially, rather than federal plants, which ship their products inter-provincially and internationally. Commodity class is an important factor, which must be accounted for in a food animal syndromic surveillance system. Older animals are not only more likely to be condemned due to an increased incidence of disease, but are more likely to be processed in certain abattoirs that specialize in this animal class. Consequently, accounting for animal class is important in determining the expected or baseline condemnation rates at an abattoir.

Economic factors, such as commodity sales price, also appear to play a role in condemnation rates, as we found that condemnation rates in cattle were higher when the sales price was above the yearly median. This may be due to a difference in the quality of animals being sent to slaughter depending on the sales price. If the price were too low, the cost of shipping animals of questionable quality may have exceeded the potential return for the producer. There was also an association found between condemnation rates and audit rating. Condemnation rates tended to be higher in C- rated abattoirs compared to those abattoirs with higher ratings. This may be due to certain abattoirs accepting a larger proportion of older or poorer quality cattle; however, there were only a small number of C-rated abattoirs during the study period. The unrated abattoirs were unlikely to have influenced the model since the condemnation rates for abattoirs in this category were not significantly different from abattoirs with other ratings. These results demonstrate the need to consider not only biological factors, but also non-biological factors, which may be associated with condemnation rates.

This study did not find a statistical association between condemnation rates and factors dealing with abattoir throughput. However, it is also important to consider abattoir characteristics, such as the number of weeks and/or the number of animals processed at abattoirs. There was great variability in the number of weeks and the number of animals processed in Ontario provincial abattoirs, which may affect quantitative data analysis. For instance, a spatio-temporal surveillance method such as the spatio-temporal scan statistic using a space-time permutation model assumes the background population remains relatively stable over time [24]. This is not the case in Ontario provincial abattoir data, as only 19% of abattoirs consistently process cattle throughout the year. If the background population increases or decreases faster in certain areas, there is a risk of population shift bias, which can cause biased p-values [24]. This bias may cause abattoirs to be identified in a high rate cluster solely due to the irregular timing of processing at certain abattoirs, not due to a true disease outbreak. It may be more appropriate to select sentinel abattoirs from regions throughout Ontario, which have a more consistent and stable processing capacity throughout the year to more accurately capture disease clusters among abattoirs.

It is important to understand the factors affecting and even biasing abattoir surveillance data, before any quantitative methods can be chosen in the design of a surveillance system. This study identified and discussed the implications of biological and non-biological factors, which may affect quantitative surveillance methods. Once these factors are identified, appropriate adjustments can be made to the quantitative methods being used for outbreak detection. For instance, a study by Kleinman et al. [15] compared the performance of the space-time scan statistic using unadjusted data for lower respiratory complaints and model-adjusted data for day of week, month, holidays and local history of illness. The study found significantly lower false detection rates in the model-adjusted analysis compared to the unadjusted.

## Conclusions

This study has identified various seasonal, secular, biological and non-biological factors that may be associated with the expected incidence of disease and indicates the potential importance of adjusting for these factors when applying quantitative methods for any disease surveillance system. Specifically, this study found that animal class, year, season, price, and audit rating impact condemnation rates from provincially inspected cattle abattoirs in Ontario. Similarly, the quality of spatial data should also be part of the assessment of potential biases that may arise from surveillance data prior to decisions concerning the application of spatial and/or spatial-temporal surveillance techniques.

## Authors' contributions

GDA performed the statistical analysis and drafted the manuscript. WBM was involved in the acquisition of data, and the drafting and revising the manuscript for intellectual content. DLP and OB were involved in the conception and design, analysis and interpretation of data, and revising manuscript critically for important intellectual content. KGB was involved in the interpretation of data, and the revising of the manuscript critically for important intellectual content. All authors read and approved the final manuscript.
